# Patterns of renal disease in South Korea: a 20-year review of a single-center renal biopsy database

**DOI:** 10.1080/0886022X.2017.1348955

**Published:** 2017-07-19

**Authors:** Ho Sik Shin, Dae Hyeon Cho, Soo Kyoung Kang, Hyun Jeong Kim, Soo Young Kim, Joung Wook Yang, Gyong Hoon Kang, Ye Na Kim, Yeonsoon Jung, Bong-kwon Cheon, Hark Rim

**Affiliations:** a Department of Internal Medicine, Kosin University College of Medicine, Busan, Korea;; b Department of Pathology, Kosin University College of Medicine, Busan, Korea

**Keywords:** Renal disease, renal biopsy, IgAN, MsPGN, MGN

## Abstract

**Background:** Several registries and centers have reported the results of renal biopsies from different parts of the world. As there are few data regarding the epidemiology of glomerulonephritis (GN) in South Korea, we conducted this study on renal biopsy findings during the last 20 years from a single center.

**Methods:** Data for 818 patients who underwent renal biopsy at our center between 1992 and 2011 were collected retrospectively. All kidney specimens were examined with light microscopy (LM) and immunofluorescent microscopy (IF).

**Results:** There were 818 cases of native kidney biopsies. In cases of primary GN, the most frequent type of renal pathology in adults (18–59 years) was mesangial proliferative GN (MsPGN, 34.5%) followed by IgA nephropathy (IgAN, 33.3%) and membranous GN (MGN, 8.8%). Indications in adults (18–59 years) were asymptomatic urinary abnormalities (75.3%) followed by nephrotic syndrome (19.8%) and acute kidney injury (AKI, 3.4%).

**Conclusions:** Among 818 renal biopsy specimens, MsPGN and IgAN were the most frequent biopsy-proven renal diseases. MGN was the third most common cause of primary GN and lupus nephritis (LN) was the most common secondary glomerular disease. Our data contribute to the epidemiology of renal disease in South Korea.

## Introduction

Renal biopsy has become one of the cornerstones of nephrology practice and is an important means of diagnosing, providing prognosis and guiding treatment of many renal diseases [1]. The first description of a technique to perform a percutaneous renal biopsy was published by Rober P. Ball in the 1930s [2]. In the 1950s, a more practical and efficient technique was described by Iversen and Brun [3]. With the introduction of the Franklin-modified Vim-Silverman needle in 1954, obtaining kidney tissue for proper histological diagnosis improved by 96–98% [4–6]. Today, most hospitals perform percutaneous renal biopsy using real-time ultrasonography and automated percutaneous devices [7–9]. This technique has improved safety and increased the number of procedures that can be performed.

Several noninvasive approaches for identifying early renal damage have been proposed for the evaluation of urine or plasma biomarkers [10]. However, the impact of these biomarkers on long-term outcomes still awaits validation for use in everyday clinical practice.

The epidemiology of biopsy confirmed renal disease provides useful information about the prevalence of renal disease and its clinical manifestations. The prevalence of renal disease differs according to the period, geographic area, race, age and indications for renal biopsy [11]. Although diabetes and hypertension are the most frequent causes of chronic kidney disease (CKD), recent evidence indicates that the number of patients starting chronic renal replacement therapy due to glomerular diseases is rising [12].

As there are little data regarding the changing patterns of renal diseases, we conducted this study of renal biopsy findings during the last 20 years at a single center. The aims of this study were to provide a comprehensive report of the relative frequencies of kidney diseases according to clinical presentation and histological diagnoses and to evaluate changing patterns of renal diseases over the past 20 years.

## Methods

### Patients

The records of adult patients who underwent a renal biopsy at Kosin University Gospel Hospital, Busan, Korea, from 1 January 1992 to 31 December 2011 were retrospectively reviewed. Data from 818 patients (18–59 years (adults) and ≥60 years (older patients)) who underwent renal biopsy in our center were collected. Data included demographic data and renal syndrome at presentation. All biopsies were analyzed over four 5-year interval.

After patients were admitted to the hospital, clinical and laboratory examinations were completed in several days. If no contraindication was determined by the attending physician, then renal biopsy was performed immediately. Institutional Review Board approval was obtained prior to the start of the study.

### Pathological examination and diagnosis

All biopsy specimens were examined by the same group of clinicians, pathologists and technicians. Nephrologists performed renal biopsies. All kidney specimens were studied with LM, IF and electron microscopy (EM). EM was performed for select cases in which diagnosis was not definite by LM and IF. The final diagnosis was made for each patient on the basis of both clinical and histologic investigations. Pathological diagnosis was definitively made by combining the results of clinical data, laboratory examination, immunofluorescence and electron microscopy.

Indications for native renal biopsy were categorized into five groups: (i) nephrotic syndrome (NS), (ii) asymptomatic urinary abnormalities (AUA), (iii) acute nephritic syndrome, (iv) chronic GN, (v) AKI and (vi) systemic disease. NS was defined as proteinuria ≥3500 mg/day associated with hyperlipidemia, hypoalbuminemia and edema. AUA was defined as subnephrotic proteinuria and/or hematuria with no clinical symptoms or signs. Acute nephritic syndrome was defined as the abrupt onset of hematuria, hypertension, edema, oliguria and reduced glomerular filtration rate (GFR). Chronic GN was defined as irreversible and progressive glomerular and tubulointerstitial fibrosis, ultimately leading to a reduction in the GFR and retention of uremic toxins. Proteinuria was defined as more than 1 g of protein present in urine per day. Incomplete records, inadequate biopsies and the second biopsy in rebiopsy patients were excluded from the analysis. Pathologic results were categorized according to the age of the patients at the time of renal biopsy, that is, 18–59 years (adults) and ≥60 years (older patients). Contraindications for renal biopsy included a solitary or ectopic kidney, horseshoe kidney, uncorrected bleeding disorder, severe uncontrolled hypertension, renal infection, renal neoplasm, hydronephrosis, end-stage renal disease, congenital anomalies, multiple cysts or an uncooperative patient.

Primary glomerular diseases (PGN) included minor changes, IgAN, MsPGN, MGN, minimal change disease (MCD), focal segmental glomerulosclerosis (FSGS), membranoproliferative glomerulonephritis (MPGN) and crescentic glomerulonephritis (CGN). Secondary GN included LN, Henoch–Schonlein purpura (HSP), postinfectious GN, amyloidosis, hypertensive nephrotic syndrome (NS), pauci-immune GN, paraproteinemic disorder, hemolytic uremic syndrome (HUS) and diabetic nephropathy (DN).

## Data analysis

Data are expressed as means ± standard deviations. Discrete variables were compared with categorical and continuous variables by chi-square and *t*-tests, respectively. The distribution of patients with varying renal biopsy diagnoses between the 5-year interval was calculated using Pearson’s chi-square analysis. A *p* values <.05 was defined as significant. Statistical analyses were performed using the Statistical Package for the Social Sciences version 18.0 (SPSS Inc., Chicago, IL).

## Results

Data for 818 patients who underwent renal biopsy between 1992 and 2011 were retrospectively investigated. The average age of the patients was 37.2 years and ranged from 18 to 83 years. The male-to-female ratio was 1.2:1. The adult group (18–59 years) included 758 cases, and the older group (≥60 years) included 60 cases.

Table 1 shows the clinical diagnosis of kidney biopsy according to age. Hematuria (≥3 red blood cells/high power field) was the most common clinical diagnosis of kidney biopsy in adults (41.2%) and proteinuria was the second in adults (39.1%). However, in the older patients, proteinuria was the most common clinical diagnosis for kidney biopsy (28.3%) followed by hematuria (25.0%).

**Table 1.** Clinical diagnosis of kidney biopsy according to age.

Diagnoses	18–59 years (*N* = 758 (%))	≥60 years (*N* = 60 (%))
Proteinuria	39.1	28.3
Hematuria	41.2	25.0
Edema	14.5	23.3
Acute kidney injury	4.0	21.7
Chronic kidney injury	0.3	1.7
Hypokalemia	0.4	0
Purpura	0.4	0
Toxin	0.2	0

Information regarding pathologic distributions of native kidney biopsy according to age is summarized in Table 2. PGN was the most common pathologic distribution in all groups (adults: 81.9%, older patients: 71.1%) followed by minor changes (adults: 11.1%, older people: 13.3%).

**Table 2.** Pathologic distribution of native kidney biopsy according to age.

Diagnoses	18–59 years (*N* = 758 (%))	≥60 years (*N* = 60(%))
Primary glomerular disease	81.9	71.7
Secondary glomerular disease	5.3	10
Hereditary nephritis	0.5	0
Normal or minor change	11.1	13.3
Tubulointerstitial disease	1.1	5.0
Others	0.1	0

Table 3 presents indications for native kidney biopsy according to age. AUA was the most common indication in adults (75.3%) and older patients (48.3%). NS was second in all groups (adults: 19.8%, older patients: 30.0%).

**Table 3.** Indications for native kidney biopsy according to age.

Indications	18–59 years (*N* = 758 (%))	≥60 years (*N* = 60 (%))
Nephrotic syndrome	19.8	30.0
AUA	75.3	48.3
Acute nephritic syndrome	0.5	1.7
Chronic GN	0.3	0
AKI	3.4	20.0
Systemic disease	0.7	0

AKI: acute kidney injury; AUA: asymptomatic urinary abnormality; GN: chronic glomerulonephritis.

Table 4 shows primary glomerular diseases according to age. In all groups, MsPGN was the most common. The second most common was IgAN followed by MGN in adults. However, MGN was the second most common in older patients.

**Table 4.** Primary glomerular disease according to age.

Diagnoses	18–59 years; *N* = 621 (%)	≥60 years; *N* = 43 (%)
IgA nephropathy	33.3	10.3
MsPGN	34.5	23.1
MCD	4.2	5.1
MGN	8.8	17.9
FSGS	3.5	12.8
MPGN	7.9	5.1
Crescentic GN	0.9	10.3
Chronic GN	3.1	10.3
Minor change	4.2	5.1

FSGS: focal segmental glomerulosclerosis; GN: Glomerulonephritis; MCD: minimal change disease; MGN: membranous glomerulonephritis; MPGN: membranoproliferative glomerulonephritis; MsPGN: mesangial proliferative glomerulonephritis.

Table 5 shows secondary glomerular diseases according to age. LN was the most common secondary glomerular disease in adults (57.5%) followed by amyloidosis in adults (12.5%).

**Table 5.** Secondary glomerular disease according to age.

Diagnoses	18–59 years; *N* = 40 (%)	≥60 years; *N* = 6 (%)
Lupus nephritis	57.5	0
HSP	10.0	16.7
Postinfectious GN	7.5	33.3
Amyloidosis	12.5	0
Hypertensive NS	0	16.7
Pauci-immune GN	0	33.3
Paraproteinemic disorder	5.0	0
HUS	5.0	0
Diabetic nephropathy	2.5	0

GN: glomerulonephritis; HSP: Henoch–Schonlein purpura; HUS: hemolytic uremic syndrome; NS: nephrotic syndrome.

Table 6 shows pathologic distributions of hereditary nephritis based on age. Thin basement membrane disease was the most common hereditary nephritis.

**Table 6.** Pathologic distribution of hereditary nephritis according to age.

Diagnoses	18–59 years; *N* = 5 (%)	≥60 years; *N* = 0 (%)
Thin BM disease	100	0

BM: basement membrane.

**Figure 1.**
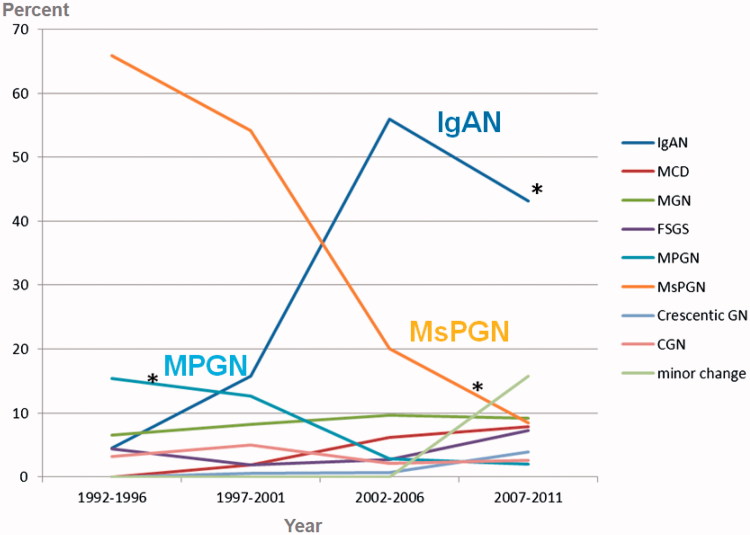
Changing trends in primary glomerular disease. ∗*p* < .05 compared with the three other time intervals. MsPGN: mesangial proliferative glomerulonephritis; MCD: minimal change disease; MGN: membranous glomerulonephritis; FSGS: focal segmental glomerulosclerosis; MPGN: membranoproliferative glomerulonephritis; CGN: chronic glomerulonephritis.

Figure 1 shows changing trends in primary glomerular disease. The relative frequency of IgAN increased significantly from 5.0% in the first quartile to 43.0% in the last quartile. The relative frequency of MsPGN decreased significantly from 63.8% in the first quartile to only 7.2% in the last quartile. IgAN was the most common primary glomerular disease since 2002. MsPGN and IgAN were the most frequent biopsy-proven renal diseases and lupus nephritis was the most common secondary glomerular disease. In the 5-year quartile comparison, the relative frequency of IgAN increased, while the relative frequency of MsPGN and MPGN decreased significantly during the past 20 years.

## Discussion

In the 5-year quartile comparison, the relative frequency of IgAN increased, while the relative frequency of MsPGN and MPGN decreased significantly during the past 20 years. MsPGN and IgAN were the most frequent biopsy-proven renal diseases, and lupus nephritis was the most common secondary glomerular disease.

The rate of kidney biopsies in numerous studies was variable because of different factors [12]. First, there were different time frame of papers [13,14]. In our study, the average age of the patients was 37.2 years and ranged from 10 to 83 years and we conducted this study on renal biopsy findings during the last 20 years (from 1 January 1992 to 31 December 2011). Second, there were lack of kidney biopsy data collection, so data may not show the situation of a given country. Third, the generalizability of data might be affected because of reference source population. Fourth, heterogenity of indications of renal biopsy. Lastly, there were socioeconomic status. There were lower biopsy rate because of the economic problem [15].

In our study, AUA was the most common indication for biopsy in adults (75.3%), and older patients (48.3%). NS was the second most common in all groups (adults: 19.8%, older patients: 30.0%). NS is the most frequent indication for renal biopsy in adults [16]. But, the usefulness and timing of renal biopsy in urinary abnormalities, diabetes, AKI or CKD of unknown origin, still have a debate. Urinary abnormalities emerged as the most common reason for performing renal biopsy in two national registries [17–19] and in two macroregional reports [15,20]. The usefullness of kidney biopsy in patients with isolated non-nephrotic proteinuria is not known. According to the patient’s age, there are different in the diagnostic approach to isolated microscopic hematuria (IMH) [21]. IMH is usually associated with hypercalciuria (30–35%), hyperuricemia (5–20%) and glomerular disease in children [22]. Nonglomerular causes (such as nut-cracker syndrome, lithiasis or neoplastic disease, etc.) should be excluded in adult [21]^,^ [23]. If AKI patients had an unknown origin of AKI, AKI duration of more than 3 or 4 weeks [24], or the presence of extra-renal manifestations, suggestive of a systemic disease, non-evidence-based biopsies were usually performed. In our study, AKI was the third most common indication in the elderly (21.4%).

In our study, MsPGN was the most common primary GN. The second most common was IgAN in adult group. However, in older patients, MGN was second most common. IgAN represents the most frequent primary, biopsy-proven GN in six out of eight national registries (Italy, Spain, Czech Republic, Denmark, Scotland, Japan) [17–19,25–30], in three macroregions (Western France, Finland, Victoria-Australia) with a range of percentages for total diagnoses, and in seven single-center databases [14,31–36]. The high prevalence may be related to genetic background, since there is evidence that IgAN is linked to a gene on chromosome 6q22–23 [37]. Variations in detection rates are also reflected by regional differences in the recognition of asymptomatic microscopic hematuria or the frequency of renal biopsy. In countries where systematic screening for urinary abnormalities is performed, IgAN is the most frequent primary GN [38]. The most frequent histological patterns related to primary NS in adults are MGN, FSGS and MCD [39]. However, cases of NS were also found to be due to diabetes, systemic lupus erythematosus (SLE), infections, multiple myeloma, amyloidosis or neoplasias [40]. According to the published pathological data of renal biopsy, the distribution spectrum of kidney disease varies throughout the world. In Europe, Oceania and Asia, IgAN is the most common glomerulopathy [25,41–43]. In the United States and Brazil, FSGS is the most common glomerulopathy [44–46]. In Europe, the detection rate of MPGN has tended to drop over time [47]. In the United States, FSGS has tended to rise [48].

In our study, LN was the most common secondary glomerular disease in adults (57.5%) followed by amyloidosis in adults (12.5%). The most frequent secondary GN was lupus nephritis in Spain (8.8%) [27], Italy (2.6 p.m.p./year) [17,25], Brazil (9.8%) [46], Bahrain (15.7%) [49], Australia (13.9%) [13], Romania (7.4%) [50], Korea (8.7%) [34], China [36] and Hong Kong (20.5%) [31].

In our study, the relative frequency of IgAN increased significantly from 5.0% in the first quartile to 43.0% in the last quartile. The relative frequency of MsPGN decreased significantly from 63.8% in the first quartile to only 7.2% in the last quartile. The relative frequency of MPGN decreased significantly during the past 20 years (from 15.4% to 2.0%). IgAN was the most frequent primary glomerular disease since 2002. In this study, MsPGN tended to drop gradually. Mesangial proliferation of many causes should not be regarded as a specific lesion. With this increasing knowledge, the proportion of MsPGN to renal biopsy cases has gradually dropped [32]. Over a 23-year period between 1973 and 1995, MCD and IgAN were the most common primary GN in Korean adults and children. The most common cause of NS was found to be MCD. However, this proportion decreased as the patients increased in age [11]. Recently, a paper regarding changing prevalence of glomerular diseases in Korean adults showed that in a 5-year quartile comparison, the relative frequency of IgAN increased, while the relative frequency of MCD and MPGN decreased significantly during the past 20 years [34]. Another study on the changing prevalence of glomerular diseases in Korean adults found a similar result [51]. The reason for the increased frequency of IgAN is unknown. Changing referral patterns and attitudes toward biopsy for patients with asymptomatic urinary abnormalities are more likely explanations for this increase, rather than more stable influences within a population such as its genetic composition. There have been numerous papers published regarding frequency, histopathological analysis and clinical data of biopsy-proven kidney diseases; however, it is not always easy to compare them due to differences in indications of kidney biopsy. More subclinical IgAN cases have been reported from countries where urinalysis is included in screening programs and asymptomatic urinary abnormalities are considered an indication of kidney biopsy [52]. We also observed a decline in the relative frequency of MPGN, which is common in adults, particularly in countries with a lower socioeconomic status. An alteration in the immune balance of the T helper 1 and 2 subsets has been proposed to explain the predilection for MPGN in developing and poor nations [53]. This could also be explained by the decreased incidence of chronic bacterial infections, improved control of viral hepatitis B or C, and better socioeconomic conditions in developed countries [52].

The rate of kidney disease in older patients is higher.[ [54]. The causes of structural and functional changes of the aging kidney are age and systemic disease (diabetes, hypertension and obesity). That causes can lead to glomerulosclerosis, tubulo-interstitial fibrosis and atrophy, which makes the elderly prone to develop CKD [55–57]. Kidney biopsy in elderly patients cannot differentiate between chronic renal damage and age-related changes. But, pathologic confirmation may be required for a diagnosis, etiological frame-working and treatment [58]. AKI (12–73%) and NS of rapid onset (13–68%) are the two most common indications for renal biopsy in elderly patients [12].

Because the manifestations of renal disease in the older patients occasionally differ from the patterns observed in other age groups [59–61], we suggest that renal biopsy should be performed on an individual basis in order to improve prognosis by providing an accurate diagnosis and enabling the initiation of specific treatment.

Our study has a number of limitations. First, data were obtained from a retrospective database of tertiary care hospitals. Second, we did not evaluate hepatitis-related renal disease. Third, the number of patients diagnosed with nephrotic syndrome was small. Although renal biopsy indications did not change over the 20-year period, some patients with kidney diseases refused renal biopsy fearing its complications; therefore, the detection rates of certain pathological types had some extent of inherent bias.

## Conclusions

MsPGN and IgAN were the most frequent biopsy-proven renal diseases, and lupus nephritis was the most common secondary glomerular disease. In the 5-year quartile comparison, the relative frequency of IgAN increased, while the relative frequency of MsPGN and MPGN decreased significantly during the past 20 years.
